# Modeling the role of environmental variables on the population dynamics of the malaria vector *Anopheles gambiae* sensu stricto

**DOI:** 10.1186/1475-2875-11-271

**Published:** 2012-08-09

**Authors:** Paul E Parham, Diane Pople, Céline Christiansen-Jucht, Steve Lindsay, Wes Hinsley, Edwin Michael

**Affiliations:** 1 Grantham Institute for Climate Change, Department of Infectious Disease Epidemiology, Imperial College, London, W2 1PG, UK; 2 Department of Infectious Disease Epidemiology, Imperial College, London, W2 1PG, UK; 3 Disease Control and Vector Biology Unit, London School of Hygiene and Tropical Medicine, London, WC1E 7HT, UK; 4 Department of Biological Sciences, University of Notre Dame, Notre Dame, IN, 46556-0369, USA

**Keywords:** Malaria, *Anopheles gambiae* s.s., Temperature, Rainfall, Density-dependence, Mathematical modeling, Climate change

## Abstract

**Background:**

The impact of weather and climate on malaria transmission has attracted considerable attention in recent years, yet uncertainties around future disease trends under climate change remain. Mathematical models provide powerful tools for addressing such questions and understanding the implications for interventions and eradication strategies, but these require realistic modeling of the vector population dynamics and its response to environmental variables.

**Methods:**

Published and unpublished field and experimental data are used to develop new formulations for modeling the relationships between key aspects of vector ecology and environmental variables. These relationships are integrated within a validated deterministic model of *Anopheles gambiae* s.s. population dynamics to provide a valuable tool for understanding vector response to biotic and abiotic variables.

**Results:**

A novel, parsimonious framework for assessing the effects of rainfall, cloudiness, wind speed, desiccation, temperature, relative humidity and density-dependence on vector abundance is developed, allowing ease of construction, analysis, and integration into malaria transmission models. Model validation shows good agreement with longitudinal vector abundance data from Tanzania, suggesting that recent malaria reductions in certain areas of Africa could be due to changing environmental conditions affecting vector populations.

**Conclusions:**

Mathematical models provide a powerful, explanatory means of understanding the role of environmental variables on mosquito populations and hence for predicting future malaria transmission under global change. The framework developed provides a valuable advance in this respect, but also highlights key research gaps that need to be resolved if we are to better understand future malaria risk in vulnerable communities.

## Background

Among the potential effects of climate change on human health, the impact on infectious diseases has attracted increasing attention in recent years [1]. Vector-borne diseases (VBDs) are likely to be particularly vulnerable given the poikilothermic nature of vector survival and development, as well as the effects of temperature on pathogen development. Although the link between climatic variables and transmission has attracted interest for VBDs such as dengue and schistosomiasis, the combined global mortality of these diseases is less than 7% of that due to malaria [2], and this, combined with the significant effects of climatic variables on multiple stages of the transmission cycle, has led to malaria remaining an important focus of ongoing debate regarding climate change and VBDs [3,4].

In the context of better understanding the role of weather and climate on transmission, two modeling approaches are possible. Statistical models use empirical relationships between climatic variables and past (or current) disease incidence (or prevalence) to predict future disease trends [5,6]. Mechanistic models, on the other hand, adopt a process-based approach, incorporating known biological, epidemiological and entomological relationships affecting vector and pathogen vital rates and formulating mathematically how these combine [7-9]. Both types of model have important roles to play in improving our understanding of climate-driven transmission changes, but the focus here is on exploiting the explanatory power of the latter.

A vital component in developing reliable VBD transmission models is establishing a realistic model of the vector population dynamics, yet only a few studies have explicitly modeled and parameterized the impact of climatic drivers on vector vital rates [8,10-12]. While these studies have greatly improved our understanding of the relative importance of temperature, rainfall and relative humidity (RH) on vector populations, they also highlight the need to develop a comprehensive mathematical framework for analysing how a range of environmental factors, arising at different spatial scales, combine at the level of breeding sites to affect stage-specific vector abundance in malaria-affected regions.

This work aims to provide such a framework by formulating and parameterizing environment-vector relationships through surveying and modeling relevant experimental and field data, and incorporating these relationships within a low-dimensional, deterministic mathematical framework. Model simplicity permits ease of integration into malaria transmission models and the model is calibrated and validated against longitudinal *Anopheles gambiae* abundance data from Tanzania [13]. The model also highlights where further experimental and modeling work is required to improve parameterization, in addition to developing a framework readily generalized to different *Anopheles* species and other disease vectors.

## Methods

Given that *An. gambiae* s.s. development and mortality depends on the life cycle stage and that field data available to parameterize mathematical models is often collected daily, a stage-structured, discrete-time model (with a daily time-step) is motivated. An alternative framework is based on physiological, rather than chronological, age and this has been adopted elsewhere [7,8,10]. In physiological age-structured models, progression through the life cycle is dependent on temperature conditions within a time-step and the minimum temperature for physiological development. However, while processes such as age-dependent mortality, heterogeneities in larval instars, and oviposition differences between gonotrophic cycles are more naturally incorporated within such approaches, there are several drawbacks of relevance to this article. For a general physiological age-structured model of the form

(1) $$n\left(t+1\right)=M\left(n,t\right)n\left(t\right)$$

where $n={\left(n_{1}n_{2}n_{3}n_{4}\right)}^{\text{T}}$ and **M** is the projection matrix, the high-dimensional nature of **M** increases by an order of magnitude as temperature measurements become more precise. The dependence of development on other factors (such as RH for adults) also increases the complexity of **M**, as well as making an implicit assumption about the linearity of development with temperature that is often violated. Thus, a low-dimensional approach is instead adopted here, providing a simple, structurally-parsimonious, deterministic model that more transparently illustrates the basic structure that may be built upon in future model development, is considerably easier to construct, analyse and interpret, and may be readily appended to malaria transmission models.

Immature *An. gambiae* s.s. pass through three distinct aquatic stages (eggs, larvae (instars L1 to L4) and pupae) prior to adult development. Let *n*_*i*_(*t*) represent the number of vectors in state *i* (where *i* = 1, 2, 3 and 4 refers to eggs, larvae, pupae, and adults respectively). The exposed nature of breeding sites results in considerable vulnerability to environmental influences and the impacts of rainfall, temperature, and biotic effects on immature survival and development are considered here. For immature stages, the daily survival probability *p*_*i*_ of stage *i* is assumed to be determined by (independent) factors attributable to the mean daily water temperature *T*_*W*_ (°C), cumulative daily rainfall *R*_*t*_ (mm), prolonged periods of desiccation *D* (days), and density-dependence *DD*, so that

(2) $$p_{i}=p_{i}\left(T_{W}\right)p_{i}\left(R_{t}\right)p_{i}\left(D\right)p_{i}\left(DD\right)$$

(where *i* = 1, 2, 3), while, for adults, $p_{4}=p_{4}\left(T_{A},RH\right)$where *T*_*A*_ is the mean daily air temperature (°C) and *RH* the relative humidity (%). If *n*_*i*_*(t)* represents the number of (female) *An. gambiae* s.s. in stage *i* at the breeding site at time *t*, then

(3) $$\begin{array}{c} n_{1}\left(t+1\right)=P_{1}n_{1}\left(t\right)+F_{4}n_{4}\left(t\right),\\ n_{2}\left(t+1\right)=P_{2}n_{2}\left(t\right)+G_{1}n_{1}\left(t\right),\\ n_{3}\left(t+1\right)=P_{3}n_{3}\left(t\right)+G_{2}n_{2}\left(t\right),\\ n_{4}\left(t+1\right)=P_{4}n_{4}\left(t\right)+G_{3}n_{3}\left(t\right) \end{array}$$

where *F*_*4*_ is the average number of eggs laid per day per female adult, *P*_*i*_ is the proportion of vectors surviving and remaining in stage *i* in *t* to *t +* 1, and *G*_*i*_ the proportion surviving and progressing from stage *i* in *t* to *t* + 1. To calculate *P*_*i*_ and *G*_*i*_, the expressions from [14] are used, namely $P_{i}=\left(\frac{1-p_{i}^{d_{i}-1}}{1-p_{i}^{d_{i}}}\right)p_{i}$ and $G_{i}=\frac{p_{i}^{d_{i}}\left(1-p_{i}\right)}{1-p_{i}^{d_{i}}}$(for all values of *i*), where 0 ≤ *p*_*i*_ ≤ 1 is given by (2) and *d*_*i*_ > 1 the average duration spent in stage *i*. To parameterize the model, the literature is reviewed to source relevant data, as well as using previously unpublished data, to develop, where appropriate, functional forms for *F*_*4*_, *d*_*i*_ and the components of *p*_*i*_ in (2). The resultant population model (3) is then calibrated and validated against vector abundance data from [15].

## Results and discussion

### Modelling breeding site hydrodynamics

**Figure 1:**
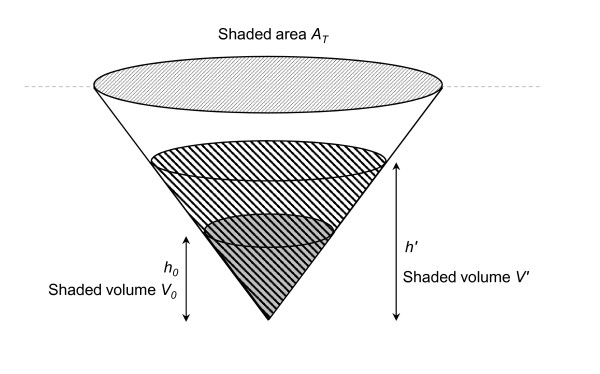
Geometry assumed for modeling breeding site hydrodynamics.

To capture the dependence of vector breeding site characteristics on environmental conditions, sites are modeled as right-centered cones to account for the increasing surface area of water available for oviposition as rainfall increases [16]. Let *V*_*t*_ be the volume of water (ml) within the site at time *t* given a fixed site opening of surface area *A*_*T*_ (mm^2^), *A’* the exposed surface area of water within the site after rainfall (mm^2^) (where *A’* ≤ *A*_*T*_) (which is then used to calculate the evaporation *E*_*t*_ from the site at the end of day *t*), and *h’* the water depth after all daily rainfall (mm) (see Figure 1). 

For $V^{'}\leq V_{\max}=\frac{1}{3}A_{T}h'/1000,$

(4) $$V_{t+1}=V_{t}+\frac{A_{T}R_{t}}{1000}-\frac{A'E_{t}}{1000}\text{,}$$

where *E*_*t*_ is the evaporation from the site on day *t* (mm). Since the total volume of water (existing volume plus new rainfall) on day *t* is $V'=V_{t}+\frac{A_{T}R_{t}}{1000}$ and $A'=1000\left(\frac{3V'}{h'}\right)\text{,}$ substituting into (4) gives

(5) $$V_{t+1}=\left(V_{t}+\frac{A_{T}R_{t}}{1000}\right)\left(1-\frac{3E_{t}}{h'}\right)\text{.}$$

To determine *h’*, consideration of the geometry of the cone before and after rainfall on day *t* gives, using similar triangles, *V’*/*V*_*0*_ = (*h’*/*h*_*0*_)^3^ (where *V*_*0*_ and *h*_*0*_ are the initial volume and depth of water respectively). Rearranging for *h’*, using the expression above for *V’*, and substituting into (5) gives

(6) $$V_{t+1}=\left(V_{t}+\frac{A_{T}R_{t}}{1000}\right)\left(1-\frac{3E_{t}}{h_{0}}{\left(\frac{V_{0}}{V_{t}+\frac{A_{T}R_{t}}{1000}}\right)}^{\frac{1}{3}}\right)\text{.}$$

To calculate *E*_*t*_, the standard FAO Penman-Monteith method is used to first calculate the daily reference crop evapotranspiration *ET*_*0*_ (mm/day) [17] as

(7) $$ET_{0}=\frac{0.408\Delta \left(R_{n}-G\right)+900\gamma U_{2}\left(e_{s}-e_{a}\right)/\left(T_{A}+273\right)}{\Delta +\gamma \left(1+0.34U_{2}\right)}\text{.}$$

Here, Δ is the slope of the vapour pressure curve (kPa°C^-1^) (which depends on *T*_*A*_), *R*_*n*_ the daily net radiation transferred to the breeding site (MJm^-2^ day^-1^) (which, for a given location and day number, depends on the daily cloud fraction *CF* (through its relationship with the number of sunshine hours per day), dew-point temperature *T*_*DP*_ (°C), minimum daily temperature *T*_*min*_ (°C) and maximum daily temperature *T*_*max*_ (°C)), *G* the soil heat flux (MJm^-2^ day^-1^), *γ* the psychrometric constant (kPa°C^-1^) (constant for a given site), *U*_*2*_ the wind speed at 2 m (ms^-1^), *e*_*s*_ the saturation vapour pressure (kPa) (dependent on *T*_*min*_ and *T*_*max*_), and *e*_*a*_ the actual vapour pressure (kPa) (dependent on *T*_*DP*_). The climatic variables *R*_*t*_, *T*_*A*_, *T*_*DP*_ and *CF* are readily available from the ECMWF ERA-40 re-analysis dataset [18], while *U*_*2*_ may be approximated from *U*_*10*_ (the wind speed at 10 m, available from ERA-40) using the conversion *U*_*2*_ = 0.748*U*_*10*_[17]. The outgoing heat conduction between the water body and surrounding soil *G* is typically negligible compared to *R*_*n*_[17] and, as in [17], is neglected here.

Daily evaporation from an exposed breeding site is likely to differ from *ET*_*0*_, however, due to differences in the reflectivity, heat capacity and typical microclimatic conditions of water bodies compared to crops. Pan evaporation *E*_*pan*_, the evaporation rate from pans filled with water and sunken into the ground, is more akin to breeding site conditions and hence *E*_*t*_ can be estimated as

(8) $$E_{t}=E_{\mathrm{pan}}=\frac{ET_{0}}{K_{p}}\text{,}$$

where *K*_*p*_ is an empirically-derived pan coefficient (dimensionless) that depends on the type of pan, breeding site surroundings, *RH* (obtained from $RH=100\exp\left(17.27T_{\mathrm{DP}}/\left(237.3+T_{\mathrm{DP}}\right)-17.27T_{A}/\left(237.3+T_{A}\right)\right)$ and *U*_*2*_. Although immature *An*. *gambiae* s.s. typically prefer clear water, examples of breeding within turbid waters also exist [19], but the turbidity of water does not typically affect *ET*_*0*_ (and hence *E*_*t*_) by more than 5% [17], so this is ignored here. Daily values of *K*_*p*_ are estimated using the empirical tables for Colorado sunken pans (with 1 m radius dry fetch) in [17] based on daily values of *RH* and *U*_*2*_. A summary of model parameters is given in Table 1. 

**Table 1:** Key model variables, parameters, and climatic variables

**Quantity**	**Definition**
*n*_*i*_(*t*)	The number of *An*. *gambiae* s.s. in stage *i* on day *t* (where *i* = 1, 2, 3, and 4 corresponds to eggs, larvae, pupae, and adults respectively)
*p*_*i*_	The daily survival probability of stage *i*
*d*_*i*_	The average duration spent in stage *i* (days)
*V*_*t*_	The volume of the breeding site on day *t* (ml)
*E*_*t*_	Evaporation from the breeding site on day *t* (mm)
*D*	The number of consecutive days without water in the breeding site (days)
*T*_*A*_	Daily mean air temperature (°C)
*T*_*W*_	Daily mean water temperature in the breeding site (°C)
*R*_*t*_	Total rainfall on day *t* (mm)
*T*_*DP*_	Dew-point temperature (°C)
*RH*	Relative humidity (%) (can be calculated from knowledge of *T*_*A*_ and *T*_*DP*_)
*CF*	Cloud fraction
*T*_*min*_	Minimum daily temperature (°C)
*T*_*max*_	Maximum daily temperature (°C)
*U*_*2*_	Wind speed at 2 m (ms^-1^)

### Environmental influences on immature development

#### Rainfall

Rainfall typically correlates strongly with vector abundance and malaria prevalence [20]. Anopheline species often differ in their habitat preference – *An. gambiae* s.s. prefer to breed in small, shallow, temporary rain pools or stagnant bodies of water fully exposed to the sun (such as hoof marks, tyre tracks or other pools created during land use changes) [21], while other species within the *An. gambiae* complex differ in their preference for freshwater, brackish and saline water [19]. To capture the dependence of oviposition behaviour on environmental conditions, let *N*_*EP*_ and *N*_*EO*_ be the number of eggs per female per oviposition produced and laid (respectively), so that

(9) $$N_{\mathrm{EO}}=f_{t}N_{\mathrm{EP}}$$

where $0\leq f_{t}\leq 1$ is the proportion of eggs laid given the environmental conditions on day *t*. *An. gambiae* s.s. oviposition may be influenced by two signals – a chemical cue directing the suitability of habitat water for oviposition and the existing density of juveniles present [22-25]. Dependence on the latter is quantified using the oviposition index *OI* introduced in [26]. Using [24] and refitting to find *OI* as a function of the number of immature per ml *ρ*_*t*_ (using data on L1 and L2 instars) gives

(10) $$OI_{t}=1.037\exp\left(-6{\left(\rho_{t}-0.317\right)}^{2}\right)-\text{0.616.}$$

It is shown in [24] that this does not depend on the number of eggs present, while [22] demonstrates that pupae presence also has no significant influence on oviposition choice. Thus, for the model here, where *n*_*i*_*(t)* represents the number of vectors in stage *i*, the relevant density is $\rho_{t}=n_{2}\left(t\right)/V_{t}$. Hence, since $OI=\left(N_{t}-N_{s}\right)/\left(N_{T}+N_{s}\right)$, where *N*_*T*_ and *N*_*S*_ are the number of eggs laid in the test substrate (pool water with larvae) and control substrate (pool water without larvae) respectively,

(11) $$f_{t}=\frac{N_{\mathrm{EO}}}{N_{\mathrm{EP}}}=\frac{N_{t}}{N_{T}+N_{S}}=\frac{1}{2}\left(OI_{t}+1\right)\text{,}$$

whereupon substituting from (10), and assuming that L3 and L4 presence has the same effect on site-attractiveness,

(12) $$f_{t}=0.51\exp\left(-6{\left(n_{2}\left(t\right)/V_{t}-0.317\right)}^{2}\right)+\text{0.192.}$$

**Figure 2:**
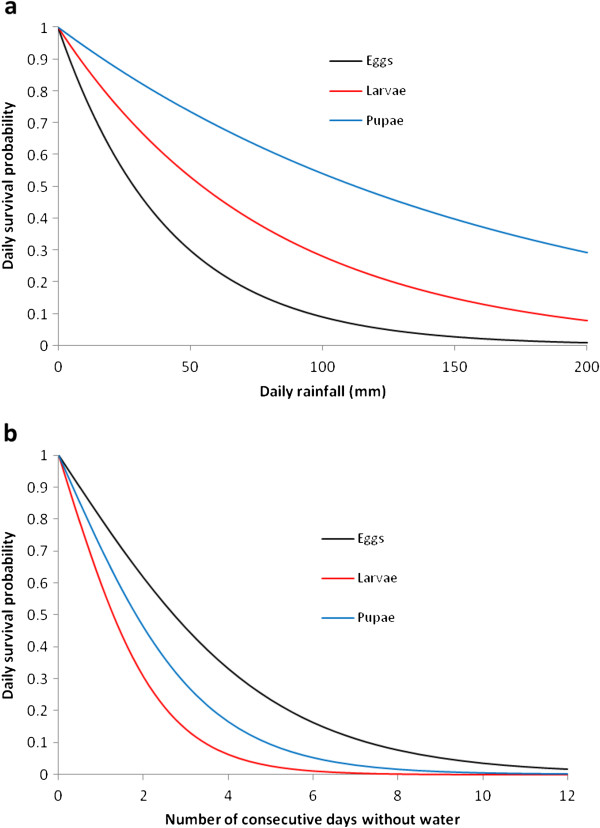
**Juvenile survival in response to (a) excess rainfall (*p***_***i***_**(*R***_***t***_**)) and (b) desiccating conditions (*p***_***i***_**(*D*)).**

In addition to creating breeding sites and influencing the characteristics of existing pools, high levels of rainfall have been associated with significant immature mortality, either due to flushing from habitats or from secondary effects [27]. These are aggregated here into total rainfall-induced mortality, modeling the decrease in survivorship by letting *p*_*i*_(*R*_*t*_) represent the daily survival probability of immatures in stage *i* given rainfall *R*_*t*_. It is assumed that $p_{i}\left(R_{t}\right)=\exp\left(-\sigma_{i}R_{t}\right)$ (*i* = 1, 2, 3) where *σ*_*i*_ quantifies the decrease in survival of stage *i*. Given the focus on L1 and L4 larvae in [27] and the absence of data elsewhere on egg and pupal mortality due to rainfall, eggs and pupae are assumed to respond similarly to L1 and L4 larvae respectively (although pupal response may differ from L4 larvae in reality due to their ventral air space that aids buoyancy, yet significantly increases mortality if this hydrostatic balance is disrupted [28]). Assuming average L1 and L4 losses of 17.5% and 4.8% per night respectively over the study period with 207 mm rainfall across 26 rainfall nights (K. P. Paaijmans, pers. comm.) gives $\sigma_{1}={0.0242mm}^{-1}$ and $\sigma_{3}={0.00618mm}^{-1}$. Given that the model here does not distinguish between larval instars, the average duration spent in each instar (as a function of *T*_*W*_) is accounted for by interpolating between L1 and L4 mortalities in [27] to determine L2 and L3 survival, whereupon averaging over all temperatures gives $\sigma_{2}={0.0127mm}^{-1}$ (Figure 2a). 

The prolonged absence of water also affects immature longevity; anopheline egg survival in desiccating conditions is two to three weeks [29], while *An. gambiae* s.l. eggs are viable for up to 12 days without water [30]. To model the decrease in egg viability in dry habitats, the findings of [31] are used, which demonstrate that the duration of exposure to desiccating conditions is a better measure of egg viability than soil moisture content. If *p*_*i*_(*D*) is the daily survival probability of stage *i* given *D* days without water, the functional form *p*_*i*_(*D*) = 2exp(−*ω*_*i*_*D*)/(1 + exp(−*ω*_*i*_*D*)) (*i* = 1, 2, 3) is fitted, where *ω*_*i*_ quantifies the sensitivity of stage *i* to desiccation and the functional form ensures that survival is near unity when *D* is small and approaches zero as desiccation increases. Least-squares estimation using field populations under medium-moisture conditions gives *ω*_*i*_ = 0.405days^−1 ^(*R*^2^ > 0.99). Survival of larvae and pupae may be similarly parameterized using [29], which demonstrates that L4 larvae survive significantly better than L1, L2 and L3 instars in such conditions – weighting by the average duration in each instar stage gives *ω*_2_ = 0.855days^−1^ (*R*^2^ = 0.97). In the absence of data on pupal survival, pupae are assumed to demonstrate a similar response to L4 larvae, whereupon using [29] gives *ω*_2_ = 0.602days^−1^ (*R*^2^ = 0.94) (Figure 2b).

#### Temperature

**Figure 3:**
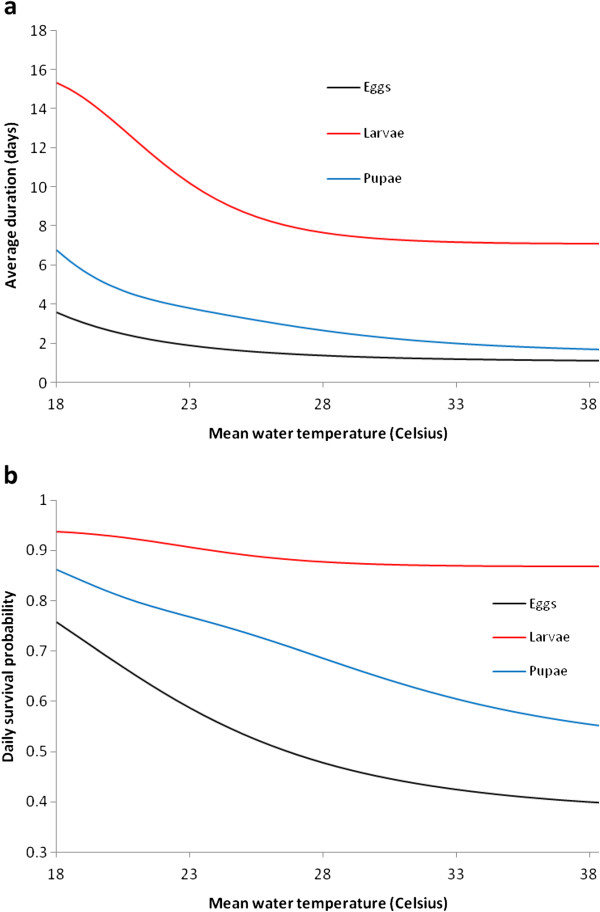
**(a) Average development time *d***_***i***_**(*T***_***W***_**) and (b) survival probability *p***_***i***_**(*T***_***W***_**) of immature stage *i*.**

Despite the strong influence of water temperature on immature populations, few detailed experimental studies have been undertaken. The model here requires the daily survival probability *p*_*i*_(*T*_*W*_) and stage duration *d*_*i*_(*T*_*W*_) for each *i*. For all three stages, age-independent mortality is assumed and hence $p_{i}\left(T_{W}\right)=\exp\left(-1/d_{i}\left(T_{W}\right)\right)$ (Figure 3a and 3b).

Egg survival is poor outside 10-40°C and [32] find that no *An. gambiae* s.s. eggs survive more than five hours at or above 41°C, with survival decreasing exponentially beyond 40°C. For egg development time *d*_*1*_(*T*_*W*_), the functional form of [33], with the corrected coefficients of Bayoh and Lindsay (unpublished data) (Table 2), is adopted. 

**Table 2:** **Average duration *d***_***i***_**(*T***_***W***_**) of immature stage *i*at water temperature *T***_***W***_**(from Bayoh and Lindsay (unpublished data))**

**Parameter**	**Functional form**
*d*_*1*_(*T*_*W*_)	$1.011+20.212{\left(1+{\left(\frac{T_{W}}{12.096}\right)}^{4.839}\right)}^{-1}$
*d*_*2*_(*T*_*W*_)	$8.130+13.794{\left(1+{\left(\frac{T_{W}}{12.096}\right)}^{4.839}\right)}^{-1}$− *d*_1_(*T*_*W*_)
*d*_*3*_(*T*_*W*_)	$8.560+20.654{\left(1+{\left(\frac{T_{W}}{19.759}\right)}^{6.827}\right)}^{-1}-d_{2}\left(T_{W}\right)$

Of the juvenile stages, larval survival demonstrates the strongest dependence on temperature and the effect of competition between *An. gambiae* s.s. and *Anopheles arabiensis* on temperature-dependent survival has been examined [34]. The relationship between survival, development and water temperature, and age-dependent mortality, for *An. gambiae* s.s. is considered in [35]. Larval duration is parameterized as a function of *T*_*W*_ in [5], but this is *An. gambiae* s.l., rather than *An. gambiae* s.s. Moreover, this parameterization is based only on temperatures between 23.0 and 32.8°C and extrapolating to temperature extremes gives inconsistent results with experimental findings in [33] (such as development times around 30 days at 18°C in the former compared to 15 days in the latter). While [10] provides a literature survey of larval development times as a function of *T*_*W*_, eight of the twelve data points for *An. gambiae* s.s. are calculated from [33] on the assumption of eggs and pupae developing within one day, which is inconsistent with experimental data in the latter. The revised coefficients from Bayoh and Lindsay (unpublished data) are therefore used to determine *d*_*2*_(*T*_*W*_).

Aside from the work of [36] on the effects of temperatures from 21.2 to 29.5°C on *An. gambiae* s.l. pupal mortality and [33], there is little experimental data to parameterize pupal development and survival. The latter, with the corrected values in Bayoh and Lindsay (unpublished data), are therefore used to parameterize *d*_*3*_(*T*_*W*_).

Finally, it is important to note the importance of using water temperature to calculate juvenile survival and development, rather than air temperature. The difference between mean daily water and air temperatures is typically around 3-6°C depending on factors such as breeding site dimensions, microclimate and weather conditions [32,37]. To account for this, it is assumed that $T_{W}=T_{A}+\Delta T\text{,}$where ΔT>0 is assumed to capture all thermodynamic processes taking place at breeding sites leading to a difference between mean water and air temperatures. Lower and upper temperature thresholds for juveniles are taken from [33].

#### Predation and density-dependence

**Figure 4:**
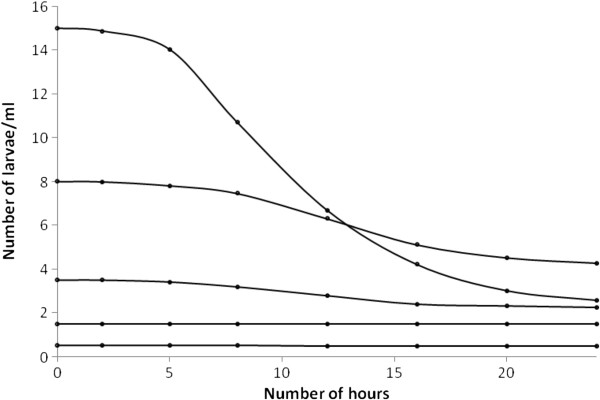
The daily survival of larvae as a function of initial density.

Density-dependent juvenile mortality arises from several sources. Body size and intra-species competition for resources, together with inter-species competition, significantly affect the population dynamics of many mosquito species and recent work has demonstrated the importance of larval density on juvenile *Anopheles* development and ecology [38]. Here, only within-stage density-dependent mortality is assumed and the potential effects of juvenile density on adult longevity or fertility are not considered. Figure 4 demonstrates the dependence of larval survival on existing larval density (H Tsila, unpublished data), while field populations of *Anopheles* larvae typically demonstrate low densities (for species that do not breed in tree holes or containers) – some field estimates suggest densities of less than 0.3/ml in rice fields, pools and small ponds [36,39], while others suggest densities around 0.02-0.06 larvae/ml and 1.5 larvae/ml [40,41] (respectively). Comparing these estimates with Figure 4 suggests that larval densities in field populations occur in regimes where intra-species competition for resources is minimal, suggesting that density-dependent mortality is most likely due to predation, although cannibalism may also occur [42]. 

Field observations also suggest the spatial aggregation of juvenile *An. gambiae* s.l., with larvae typically distributed negative binomially [43]. To model the effects of density-dependence, these observations are incorporated within application of the framework of [44] for developing first principles population models given knowledge of intra-species competition and spatial distribution. For larval populations following a negative binomial distribution and demonstrating predominantly contest competition (given the dominance of predation, also consistent with findings elsewhere such as [40]), [44] demonstrates that if *X*_*t*_ is the population size at time *t*

(13) $$X_{t+1}=bm\left(1-\frac{\lambda^{\lambda }}{{\left(\lambda +X_{t}/m\right)}^{\lambda }}\right)\text{,}$$

where *m* is the number of resource sites across which the population is distributed, *λ* the aggregation parameter of the negative binomial distribution and *b* a positive constant. The value of *λ* is calculated by averaging the aggregation parameters from the five experiments in [43] for which the negative binomial provides the best fit to obtain λ=1.5. To determine *b*, consider, without loss of generality, an arbitrary one litre volume of water within a breeding site and divide this into 1ml blocks (so m=1000). The observed difference in juvenile mortality between field data and the contribution from temperature and rainfall is attributed to density-dependence. Given that the datasets used consider survival from L1 instars and that the duration of field studies is often longer than the development time from L1 to pupae, it is assumed that predation reduces larval and pupal survival and acts identically on both stages. Since the population affected is $n_{2}\left(t\right)+n_{3}\left(t\right)$, the number of larvae and pupae per litre is $N_{23}\left(t\right)=1000\left(n_{2}\left(t\right)+n_{3}\left(t\right)\right)/V_{t}$ and $p_{i}\left(DD\right)=X_{t+1}/X_{t\text{,}}$ the daily larval and pupal survival probability due to density-dependence, *p*_*2*_(*DD*) and *p*_*3*_(*DD*), is

(14) $$p_{i}\left(DD\right)=\frac{bm}{N_{23}\left(t\right)}\left(1-\frac{\lambda^{\lambda }}{{\left(\lambda +N_{23}\left(t\right)/m\right)}^{\lambda }}\right)$$

for *i* = 2 and 3. If *n*_*2*_(*t*) + *n*_*3*_(*t*) = 0 or *V*_*t*_ = 0, *p*_*i*_(*DD*) is assumed to be unity. Predation on eggs is assumed to be negligible by comparison and anopheline rice-field survival data from [39], [41] and [45] is used to provide seven independent datasets to fit *b* at Δ*T = 3°C,* 4°C, 5°C and 6°C. For each dataset, air temperature and rainfall data from the nearest meteorological station (using [46] and where missing values are interpolated) are used to calculate the daily survival and development of larvae and pupae due to climatic influences (assuming fixed vector density and assuming no desiccation effects for rice fields) and estimate the additional mortality required to agree with the study data (attributed to *p*_*i*_(*DD*)). Two approaches are adopted, namely to (a) calculate the number of juveniles remaining after a fixed number of days (determined by the study design), and (b) track the number of cohort larvae and pupae until less than 0.05% of the original population remain. For method (a), where experimental dates are not specified, *b* is calculated for a range of plausible start dates and the average computed. No significant difference in calculating *b* using these methods is found and b=0.89 for ΔT=3°C and 0.88 for ΔT=4°C, 5°C and 6°C.

#### Environmental influences on adult development

The survival of adult *Anopheles* is sensitive to temperature and RH, although few experimental studies have examined this in detail and [11] have recently undertaken a review of parameterization work to date. Although the fitting of [47] has been used in work examining the effects of climatic variables on malaria transmission (such as [8]), this parameterization is inconsistent with [48] demonstrating that *An. gambiae* s.s. cannot survive longer than one day at 40°C. Thus, the majority of modeling studies to date investigating malaria transmission under changing environmental conditions [5,7,9,49] use

(15) $$p_{4}\left(T_{A}\right)=\exp\left(-1/\left(-0.03T_{A}^{2}+1.31T_{A}-4.4\right)\right)\text{,}$$

despite its basis on fitting a three-parameter function to three data points in the range 9-40°C [9] (with the 40°C point inconsistent with [48]). This relationship assumes no adverse effects of RH on mortality, which is unlikely given that RH < 50% leads to significantly reduced survival [50]. Field observations of *An. gambiae* adults are only approximately consistent with (18), but reflect the relatively high survival at 22-30°C [36].

**Figure 5:**
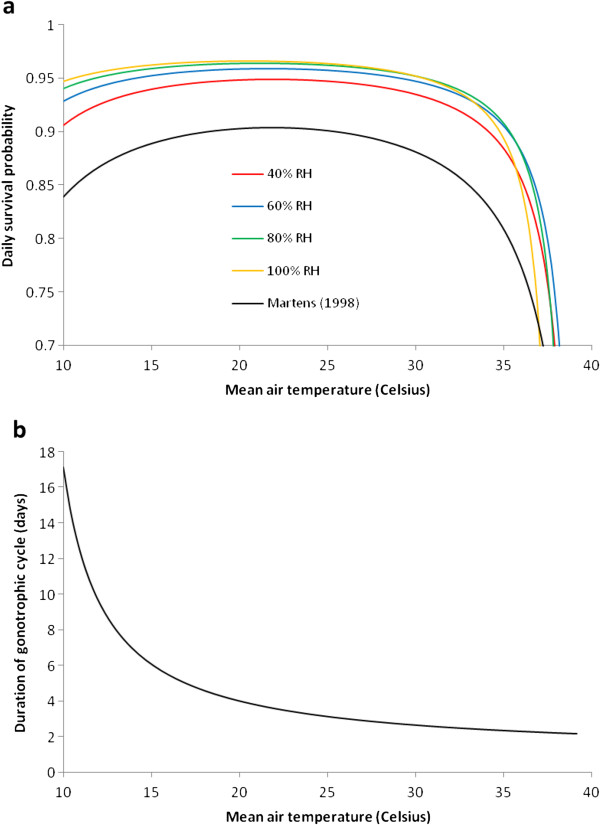
**(a) Daily (female) adult survival *p***_***4***_**(*T***_***A***_**,*RH*) versus the parameterization of**[9]**. (b)*G***_***c***_**(*T***_***A***_**) from (20).**

To obtain a more systematic fitting, experimental data from Bayoh and Lindsay (unpublished data), who estimate survival thresholds of 5°C and 40°C and, within this range, examine the effects of temperature and RH on mortality, are used. Age-independent survival is assumed and *p*_*4*_(*T*_*A*_*,RH*) fitted given mean female survival times at 5-40°C inclusive (in 5°C intervals) and 40-100% RH (at 20% intervals) to obtain

(16) $$p_{4}\left(T_{A},RH\right)=\exp\left(-1/\left(\beta_{2}T_{A}^{2}+\beta_{1}T_{A}+\beta_{0}\right)\right)$$

where $\beta_{2}=4.00\times 10^{-6}RH^{2}-1.09\times 10^{-3}RH-0.0255,$$\beta_{1}=-2.32\times 10^{-4}RH^{2}+0.0515RH+1.06$ and $\beta_{0}=0.00113RH^{2}-0.158RH-6.61$ (Figure 5a). Survival outside this temperature range is assumed to be zero, but no RH thresholds are assumed.

The duration of the gonotrophic cycle *G*_*c*_ is also temperature-dependent and [9] parameterizes this as *G*_*c*_(*T*_*A*_) = *D*_*M*_/(*T*_*A*_*T*_*M*_) where *D*_*M*_ = 36.5°C days and *T*_*M*_ = 9.9°C. An alternative functional form is given by [11] as

(17) $$G_{c}\left(T_{A}\right)=1+\frac{D_{E}}{T_{A}-T_{E}}$$

where *D*_*E*_ = 37.1°C days and *T*_*E*_ = 7.7°C. Comparing these formulations, the latter gives longer gonotrophic cycles at temperatures above 17.6°C, the regime generally of interest. On the basis of better agreement with [51], (20) is adopted (Figure 5b). To calculate *F*_*4*_, *d*_4_/*G*_*c*_(*T*_*A*_) gives the average number of ovipositions per adult across her lifetime. If the average number of eggs per oviposition is *N*_*EO*_ = *f*_*t*_*N*_*EP*_, the average number of eggs laid over her lifetime is *d*_*4*_*N*_*EO*_/*G*_*c*_(*T*_*A*_), so that *F*_4_, the average number laid per day, is

(18) $$F_{4}=\frac{f_{t}N_{\mathrm{EP}}}{G_{c}\left(T_{A}\right)}\text{.}$$

Given the absence of age-structure in this model, each gonotrophic cycle is assumed to be of equal duration for all adults and produce the same number of eggs, although studies have shown variation in both [52].

No direct influences of rainfall on adult survival are assumed (with indirect effects through changes in RH captured by (19)) and adult survival is assumed to be density-independent following [53] and the weak, but statistically significant, relationship between adult density and survivorship in [15]. There is some evidence of predation on adult *An. gambiae* s.l. at oviposition sites, with the severity potentially depending on the type of site [39], but there are few quantitative studies in this respect.

#### Model calibration and validation

To assess performance, the model is calibrated and validated against longitudinal *An. gambiae* s.l. abundance data from [13] collected in an environment free of vector controls. Data on *T*_*A*_, *T*_*DP*_ (for calculation of *RH*), (low) cloud fraction *CF*, and the horizontal and vertical components of 10 m wind speed (to calculate *U*_*2*_) are taken from the ERA-40 re-analysis dataset [18] for the rural community in Masaika, Tanzania (5 16' 0'' S, 38 49' 60'' E) (with the nearest ERA-40 point at 5^o^, 0’ 0” S, 37^o^ 30’ 0” E). Rainfall data from the Maji Depot Tanga Rainfall station (at 5 4' 58'' S, 39 5' 21'' E), approximately 35 km from Masaika, is used when available (see [13]), with missing data taken from [18]. Since the daily values of *T*_*min*_ and *T*_*max*_ are not available from [18], we derive empirical relationships between *T*_*A*_ and these variables using data from the nearest meteorological station (Tanga at 5^o^ 4’ 48” S, 39^o^ 4’ 12” E), approximately 34 km from the study site, and apply these relationships (*T*_*min*_ = 0.724*T*_*A*_ + 14.4, with *R*^*2*^ = 0.53, and *T*_*max*_ = 0.728*T*_*A*_ + 28.3, with *R*^*2*^ = 0.61) to ERA-40 data on *T*_*A*_ to estimate the associated values of *T*_*min*_ and *T*_*max*_.

**Figure 6:**
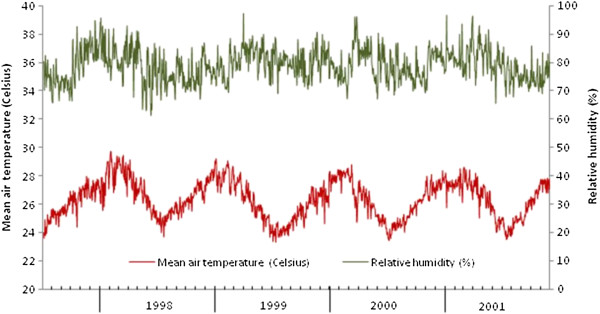
Daily air temperature and relative humidity over the calibration and validation periods.

Daily abundance data is available from 06/07/1998 to 30/11/2001 (approximately 41 months), consisting of the number of adult *An*. *gambiae* s.l. caught in CDC light traps; further details on mosquito collection and experimental procedures are given in [13]. The model is calibrated using the first twenty months of complete monthly data (August 1998 to March 2000 inclusive) and validated over the subsequent twenty months (April 2000 to November 2001 inclusive). The variation in *T*_*A*_ and *RH* over the calibration and validation periods is shown in Figure 6. Data on the average number of *An*. *gambiae* s.l. caught per trap is available for each weekday in the period (aside from short breaks for public holidays), but not at weekends. Given the daily time-step nature of the model, weekend abundance is estimated using linear interpolation and these values appended to the weekday values. This data is then aggregated by month and the model fitted at this scale. A minimum 365 day burn-in period is applied to remove early model transients. 

For model calibration, the average number of adult *An. gambiae* s.l. per light trap is fitted to model output after the burn-in period. To account for the difference in scale between data and the model, the scaled fecundity $\overset{Â¯}{F_{4}}=\alpha_{1}F_{4}$ and adult *An*. *gambiae* s.s. abundance $\overset{Â¯}{n_{4}}=\alpha_{2}n_{4}$are defined and just three parameters fitted over the calibration period – the scale parameters *α*_1_ and *α*_2_, and Δ*T*. All other parameters are derived from parameterizations in this paper and local breeding site properties (altitude and latitude). It is assumed that $N_{\mathrm{EP}}=120$ (based on model calibration in [7]) and breeding site dimensions consistent with the characteristics of typical *An*. *gambiae* s.s. habitats (in the presence of multiple *An*. *gambiae* s.l. species given the collection of multiple *Anopheles* species in data collection in [13]) reported in [21], namely *A*_*T*_ = 1.79 x 10^6^mm^2^ and *h*_*0*_ = 97mm. An initial water volume of 1 litre is assumed (*V*_*0*_ = 1000ml). Model fit to data is found to be independent of the initial conditions, so 100 mosquitoes are arbitrarily initially assumed to be in each lifecycle stage.

**Figure 7:**
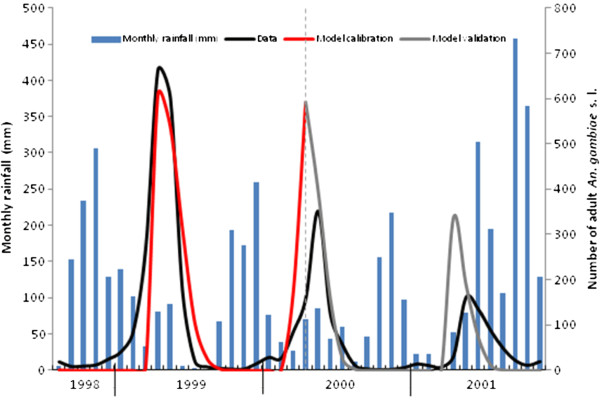
**Model calibration (August 1998 to March 2000 inclusive) and validation (April 2000 to November 2001 inclusive) versus adult *An*. *gambiae*s.l. abundance data from**[13].

**Figure 8:**
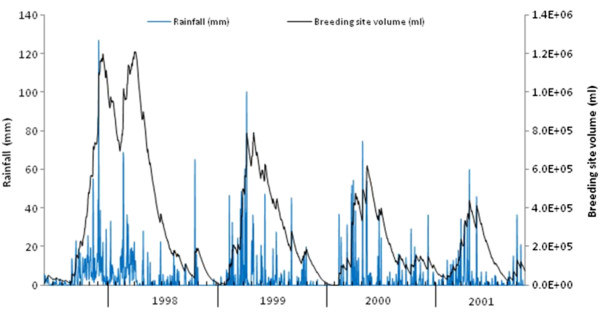
Daily rainfall and breeding site water volume over the calibration and validation periods.

**Figure 9:**
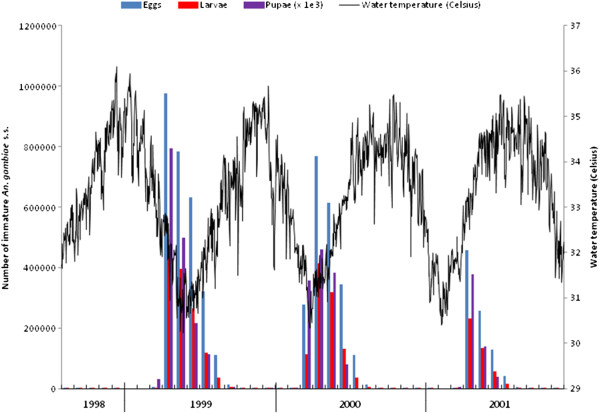
Water temperature behaviour and dynamics of the number of eggs, larvae, and pupae over the calibration and validation periods.

**Figure 10:**
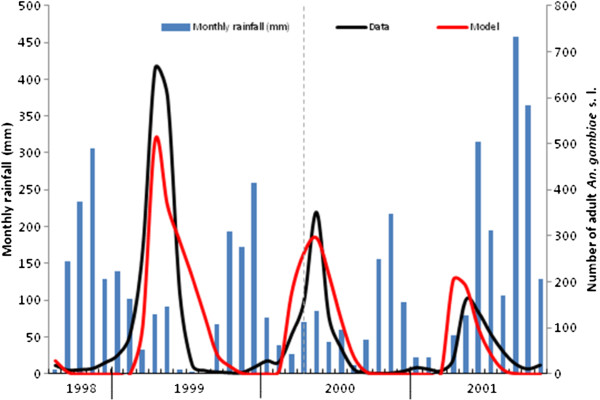
**Model fitting against all 40 (complete) months of adult *An*. *gambiae*s.l. abundance data in**[13]

Fitting the model using least-squares to the 20 month calibration data gives the best-fit parameters $\alpha_{1}=141.612$, $\alpha_{2}=0.030$ and ΔT=6.9°C (*R*^*2*^ = 0.84). Running the model for a further 20 months with these parameters and assessing the goodness-of-fit gives *R*^*2*^ = 0.50 across the validation period (Figure 7). The model is encouragingly able to capture the overall decline in *An. gambiae* s.l. abundance in Masaika reported in [13] across the calibration and validation periods, as well as the general seasonal trend (although the timing of the two abundance peaks in the validation period are underestimated by one month in both cases, as well as the magnitude of the peaks). The water volume within the breeding site over time (with these best-fit parameters) is shown in Figure 8, while the immature population dynamics, and estimated daily water temperature, are plotted in Figure 9. Alternatively fitting the model across the entire 40 months of data (Figure 10) gives *R*^*2*^ = 0.70 (with $\alpha_{1}=280.486$, $\alpha_{2}=0.026$ and ΔT=6.1°C) and, in this case, the timing of two of the three seasonal peaks are correctly predicted, as well as the approximate severity of these peaks. The fitted values of ΔT=6.9°C and 6.1°C are slightly greater than typical Δ*T* values observed in the field (values in [37], for example, lie in the range 4.0-6.1°C on clear days for three different sized pools), and this reflects the simplified nature of the $T_{W}=T_{A}+\Delta T$ formulation and fitting a single mean value of Δ*T* across annual timescales. Future refinements will improve this component of the model by calculating Δ*T* from thermodynamical principles based on daily weather conditions and this is expected to further improve model fit. Nonetheless, the model offers the potential for mechanistic insight into vector response to temperature, rainfall, RH, wind speed and cloudiness, and hence how future changes in these variables may affect mosquito dynamics. The results suggest that the observed decline in vector numbers (and malaria) reported in [13] could, in turn, be due to long-term changes in environmental conditions. Further model analysis (such as application of the methods of matrix population modeling [54]) will provide valuable insight into the dominant environmental variables influencing the observed changes in vector numbers, as well as furthering our understanding of the dominant drivers on short and long-term timescales. While such analysis is beyond the scope of this paper and will follow in a forthcoming article, these results highlight the explanatory power of validated mathematical models and their role in evaluating the effects of temporal changes in weather and climate on vector dynamics and, ultimately, disease transmission. 

## Conclusions

Along with *An. arabiensis* and *Anopheles funestus*, *An. gambiae* s.s. is one of the principal malaria vectors in Africa [19] and understanding its ecology and dynamics is vital in better understanding the associated impact on malaria transmission and the prospects for eradication [55], as well as the effectiveness of vector controls in different communities and settings. Vector population dynamics are driven by a range of biotic and abiotic factors and clarifying the role of both is key, particularly in the context of how climate change may influence the future spread and distribution of VBDs. Here, a useful framework for understanding how changes in rainfall, temperature, RH, wind speed and cloudiness (both mean values and temporal variability), and density-dependence, at breeding sites may influence vector abundance is presented. By calibrating and validating the model against longitudinal abundance data, this framework is shown to be capable of reproducing the observations in [13] on long-term timescales, suggesting a mechanistic underpinning of mosquito dynamics in terms of environmental variables, an important result given the ongoing debate regarding the link between malaria transmission and climatic changes in Africa [3,4]. This work also highlights the power of mathematical models in addressing key questions surrounding the role of environmental variables, compared to the multitude of other ecological, epidemiological, socioeconomic and demographic factors, on disease transmission [1]. An important advance of this work is the construction of a modeling framework enabling the linkage of climatic events at large spatial scales to processes at the localized scale of vector breeding sites, enabling assessments of how climatic phenomena at different scales may affect disease transmission in host communities.

Model reliability may be enhanced with improved parameterization and future experimental and modeling research will lead to further understanding of species-specific *Anopheles* population dynamics and their response to environmental variables. These include (i) improving our understanding of *Anopheles* oviposition behaviour, (ii) better quantifying the role of rainfall and temperature on egg, larval and pupal survival, as well as the role of heterogeneities, such as body size, that might influence response, (iii) improved modeling of the relationship between air and water temperatures at breeding sites, (iv) improving our understanding of density-dependent effects on juvenile and adult development and survival (including intra-specific competition, inter-specific interactions between species, cannibalistic tendencies, and predation, as well as their dependence on climatic variables), (v) assessing evidence for age-dependent mortality in juveniles and adults, and (vi) better understanding variability in gonotrophic cycles.

New longitudinal vector studies that simultaneously measure changes in environmental variables are also required to improve the validity and reliability of vector models, which will not only further our understanding of dominant factors driving mosquito dynamics, but will also improve our understanding of the implications for VBD transmission. Nonetheless, the approach here not only provides a useful framework for *An. gambiae* s.s. modeling, but its structure may be readily applied to other *Anopheles* species with suitable parameterization, as well as other vectors (such as *Aedes* or *Culex*). This will ultimately enable a better understanding of the response of a variety of VBDs to environmental change, an important question given the likely influences of weather and climate on many regions of VBD risk over the coming decades.

## Competing interests

The authors declare that they have no competing interests, financial or otherwise.

## Authors’ contributions

PEP conceived of the study design and framework, directed the research and wrote the manuscript. DP and CCJ both made significant contributions to the parameterization work, components of the model, and data fitting. SL contributed multiple unpublished datasets and parameterization work. WH contributed computational assistance in multiple phases of the project. EM contributed to the concept of the study. All authors read and approved the final manuscript.
